# Changes in Trauma-Related Cognitions and Emotions After Eliciting Moral Elevation: Examining the Effects of Viewing Others' Virtuous Behavior on Veterans With PTSD

**DOI:** 10.3389/frhs.2021.831032

**Published:** 2022-02-04

**Authors:** Adam P. McGuire, Joanna G. Fagan, Binh An N. Howard, Annika I. Wurm, Yvette Z. Szabo

**Affiliations:** ^1^VISN 17 Center of Excellence for Research on Returning War Veterans, Waco, TX, United States; ^2^Central Texas Veterans Health Care System, Temple, TX, United States; ^3^Department of Psychology and Counseling, The University of Texas at Tyler, Tyler, TX, United States; ^4^Department of Health, Human Performance and Recreation, Baylor University, Waco, TX, United States

**Keywords:** experimental design, veterans, guilt, shame, post-traumatic cognitions, positive emotion

## Abstract

Moral elevation is described as feeling inspired after witnessing someone perform a virtuous act. Past work suggests the features of moral elevation may be contrary to PTSD, yet few studies have directly tested its impact on relevant symptoms. This experimental study assessed changes in trauma-related cognitions and emotions from after a trauma reminder task to after an elevation induction exercise. We hypothesized that higher elevation after the induction exercise would be associated with greater reductions in cognitions and emotions. Veterans with probable PTSD (*N* = 38) completed measures of trauma-related cognitions and emotions, once after a written trauma narrative exercise (T1) and again after watching two videos designed to elicit elevation (T2). Veterans also completed measures of state elevation after each video. Results suggest veterans experienced small, significant decreases in self-blame (*d* = 0.36) and negative beliefs about others (*d* = 0.46), and medium, significant decreases in guilt (*d* = 0.68), shame (*d* = 0.60), and negative beliefs about self (*d* = 0.69) between T1 and T2. As hypothesized, higher elevation was associated with significantly greater reductions in multiple outcomes above and beyond the effects of general positive affect. Specifically, there were medium effects for changes in shame (β = −0.42, *SE* = 0.17, *p* = 0.019, Δ*f*^2^ = 0.25), negative view of others (β = −0.34, *SE* = 0.16, *p* = 0.044, Δ*f*^2^ = 0.20), and a large effect for changes in negative view of self (β = −0.31, *SE* = 0.13, *p* = 0.019, Δ*f*^2^ = 0.54). These findings suggest elevation may be well-suited to target trauma-related symptoms and future research should further examine its clinical utility.

## Introduction

For United States veterans, post-traumatic stress disorder (PTSD) is a common consequence following exposure to a traumatic event that is associated with distress, impaired functioning and increased healthcare utilization (1–3). Symptoms of PTSD can involve distressing memories, strong negative beliefs, avoidance behaviors, higher physiological reactivity, and feelings of anger, guilt, or shame (4). Compared to civilians, service members are disproportionately diagnosed with PTSD (5). Furthermore, veterans often experience severe symptomology and poor prognoses (6), highlighting the need to study and identify new avenues to enhance treatment. The purpose of this experimental study is to examine the role of moral elevation, a positive psychology construct, in the context of experiencing trauma-related cognitions and emotions among a sample of veterans with PTSD symptoms.

### Moral Elevation

Moral elevation (hereafter, elevation) is a multi-faceted social and emotional state that typically arises after observing another person engage in virtuous behavior (7), such as an act of generosity, compassion, or perseverance. In response, people often report subjective experiences of feeling uplifted, moved, or touched, along with physiological reactions such as a lump in one's throat, piloerection (i.e., goosebumps), tears, or warmth in the chest. Subsequently, the observer may also feel motivated or inspired to imitate the virtuous behavior they witnessed (8, 9).

Previous studies have identified a wide range of benefits and correlates of elevation. For example, in non-clinical populations, elevation was found to be associated with volunteerism and community engagement (10). It has also been correlated with increased pro-social behavior (11) both in the context of considering one's own moral self-image [i.e., helping after affirming positive views of self; (12)] and for extrinsic, altruistic purposes [i.e., helping others without expectation of reward; (13)]. Additionally, elevation experiences are correlated with increased positive affect, compassionate motives, and affiliation with others (14). Notably, these studies also demonstrate the ability to induce elevation by asking participants to watch videos of real people demonstrating virtuous behavior—a common method in elevation research (8, 13, 14). Thus, previous research suggests elevation has the capacity to affect emotional states, as well as motivations and behaviors associated with social engagement by means of compassion, pro-sociality, or social connection more broadly. However, it should be noted that many of these studies used community or university samples and the effects of elevation on military and clinical populations are not well-understood.

### Elevation and PTSD

Despite limited research on clinical populations, there is some initial support for the notion that elevation could be useful in mitigating psychological distress. Specifically, preliminary findings regarding elevation and trauma distress suggests elevation may be particularly well-suited to target the cognitive and emotional symptomatology of PTSD. Boosting moral elevation to target trauma distress warrants further investigation because it has been argued that elevation and its features are antithetical to PTSD symptoms (15). For example, military-related trauma often corresponds with exaggerated negative cognitions about the world (16), and sometimes moral injury—witnessing or perpetrating an act that violates deeply held morals or beliefs and results in significant psychological, behavioral, and spiritual consequences (17). In contrast, elevation is associated with positive appraisal of others after witnessing and perceiving virtuous acts. People suffering from PTSD may experience strong negative emotions or sometimes a numbing of positive emotions (18), whereas elevation is described as a strong positive emotional response that can also include involuntary physiological reactions (e.g., lump in throat, piloerection, etc.). Finally, individuals with PTSD often engage in strong avoidance and isolation behaviors, retreating from the world and those around them (19, 20), whereas elevation may elicit the desire to help and connect with others and behave compassionately.

To date, only a few studies have empirically examined the theorized benefits of elevation in the context of trauma-related distress. Tingey et al. (21) first demonstrated the relevance of elevation with a subclinical sample, finding that elevation was a predictor of post-traumatic growth and healthy adaptation following a college campus shooting. Expanding to a clinical population of veterans with PTSD, McGuire et al. (15) examined veterans who were enrolled in residential trauma treatment. In this pilot study, weekly measures of elevation in response to the acts of fellow group members was associated with greater weekly engagement in the group treatment and lower avoidance symptoms at post-treatment. Notably, results from the initial version of the current experimental study (Study 1) demonstrated that veterans who reported elevated levels of PTSD symptoms can indeed experience elevation in response to portrayals of virtuous behavior (22). In Study 1, veterans reported higher elevation after they watched videos intended to induce elevation compared to a control group that was presented with videos intended to induce general positive affect and amusement. Additionally, veterans in the elevation condition described other clinically-relevant outcomes in qualitative response including lessened trauma-related thoughts and emotions, feeling inspired, a desire to engage in pro-social behaviors, an urge to reach out to family members or connect with others, and a desire to be a better person. These early findings, combined with theoretical rationale, suggest that elevation might have the capacity to counteract trauma distress and PTSD symptoms; however, more research is needed to test this theory in a controlled setting with veteran participants who are suffering from significant PTSD symptoms.

### Changes in Trauma Cognitions and Emotions

Two specific symptoms of PTSD that could be influenced by elevation are trauma-related cognitions and emotions. As previously noted, negative cognitions and emotions are prominent features of PTSD that can cause significant distress (23–25). Trauma-related cognitions can include negative beliefs about the self [e.g., “There is something wrong with me as a person”; (16)], others [e.g., “People can't be trusted”; (26)], or the world in general [e.g., “Nowhere is safe”; (27)]. Two negative emotions that are prevalent among those with PTSD symptoms are guilt and shame. Guilt is considered a self-conscious moral emotion associated with feelings of remorse, and it can be a behavioral motivator to correct wrongs or return to a way of life more consistent with one's moral code (28). Shame is also a negative moral emotion but is distinct from guilt based on slightly different cognitions and outcomes; shame-based thoughts typically entail feelings of inadequacy, being damaged, or being flawed. Unlike guilt, shame is linked with acts of aggression or social isolation (17, 29).

In addition to the need for reducing distress, these targets are also well-suited for examining the within-person effects of elevation because research suggests they can change and are not stable over time. For example, a longitudinal study of Israeli combat veterans and ex-prisoners of war found that cognitions, specifically those pertaining to the benevolence and safety of the world, can worsen over time. Other experimental studies have replicated these findings with regard to worsening cognitions and emotions using script-driven imagery activities and other such trauma reminders (30, 31). Conversely, studies have also found that the severity of trauma-related cognitions and emotions can lessen over time in the context of experimental studies (32, 33) and during the course of treatment (34, 35). Given the capacity for cognitions and emotions to change—an innate goal of trauma-focused treatment for PTSD—the clinical utility of eliciting elevation could be demonstrated by assessing for within-person changes in trauma cognitions and emotions following an elevation induction exercise. This would expand our understanding of elevation's potential to impact survivors of trauma and people suffering from PTSD symptoms, while also adding to the literature by identifying a novel tool to alleviate cognitive and emotional distress in this population.

### Current Study

The purpose of this study is to investigate the specific effects of eliciting elevation in veterans with significant PTSD symptoms on trauma-related cognitions and emotions following a trauma reminder—expanding on preliminary findings from the initial experiment (Study 1) that demonstrated veterans with PTSD symptoms have the capacity to experience elevation (22). Based on existing research, it was hypothesized that negative emotions (shame and guilt) and negative cognitions about the self, others, and self-blame would reduce over time following a trauma reminder task. We also hypothesized that higher levels of elevation following exposure to elevation-inducing videos would predict greater decreases in negative emotions and cognitions from post-trauma reminder to post-elevation induction.

## Methods

### Participants and Procedures

Recruitment methods included mailing out recruitment letters to veterans enrolled in Veterans Health Administration (VHA) care, in-person recruitment in VHA PTSD clinics, flyers posted in VHA clinics and community sites, and direct referrals from VHA clinicians. First, veterans were screened for potential eligibility using the Posttraumatic Checklist for Diagnostic and Statistical Manual of Mental Disorders, 5th Edition [PCL-5; (36)]. Veterans with a total score of 33 or higher were viewed as experiencing significant symptoms of PTSD (37, 38) and therefore, deemed eligible to participate in the study. Other inclusion criteria included veteran status and at least 18 years of age. Exclusion criteria were active psychosis, active mania/hypomania, cognitive disorders (e.g., dementia, traumatic brain injury), and age of 90 years or older. Eligibility was assessed during a phone screen. Following the phone screen, eligible veterans scheduled an in-person appointment and provided written consent to participate in the study upon arrival to that appointment. All study procedures were approved by the local Institutional Review Board.

**Figure 1:**
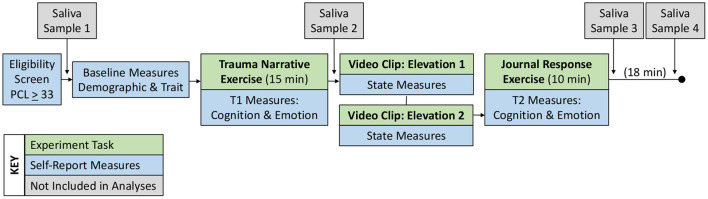
Procedures for the experimental session.

After consenting, veterans participated in an experimental session that included a series of repeated measures and study tasks. The schedule for session procedures is presented in Figure 1. First, participants completed baseline measures, then they completed a written trauma narrative describing their worst traumatic event in detail for 15 min. The purpose of this task was to elicit and measure thoughts and feelings associated with recalling a traumatic event. To ensure confidentiality of sensitive information and increase the likelihood that veterans would fully engage with the exercise, participants were notified that study staff would not collect or view their trauma narrative and participants were encouraged to destroy the written piece of paper at the end of the experimental session.

Immediately after the trauma narrative, veterans completed questionnaires that assessed the severity of trauma-related cognitions and emotions experienced at that moment (Time 1; T1). All participants were then presented with three video clips, followed by brief measures to assess state-level emotional responses to each video. The first video was a neutral stimulus that was not intended to elicit positive or negative emotions, which served as a manipulation check for elevation and positive affect responses.

Next, participants viewed two videos that were specifically intended to elicit elevation. One video described the heroic efforts of a man (self-identified as a veteran) who saved another man's life after he fell onto the subway tracks. The second video told a story about a father who competed in marathons and triathlons with his son who had paraplegia by carrying, pushing, and pulling his son through each race. The order of the videos was randomized across participants. Two videos were included with the intent to increase the *dose* of elevation experienced and increase the potential of impacting changes in trauma-related cognitions and emotions. The two elevation videos lasted ~7 min in total. In this study, there was no control condition and all participants were presented with the two elevation videos. This decision was based on two considerations. First, results from quantitative and qualitative analyses of data from Study 1 indicated the elevation videos elicited a distinct response from positive affect or general amusement that was consistent with the theory of elevation (22); thus, establishing that the same videos used in this study elicit the desired state elevation response within this population. Second, there are significant resource constraints to collecting data for an in-person, experimental study that requires a veteran sample with elevated PTSD symptom severity; thus, a within-person design without a control condition increased feasibility of completing data collection and increased power for the present set of analyses. Therefore, the present study can be considered a preliminary examination of the cognitive and emotional responses to elevation-inducing videos.

After watching both videos, participants were asked to complete a brief journal exercise describing their reactions to the video with prompts (see Supplementary Material). This exercise was added to encourage participants to reflect on what they witnessed in the elevation videos and to consider how they reacted or felt after viewing that content. After the journal exercise, they completed the same trauma-related cognition and emotion questionnaires for a second time (Time 2: T2). The two assessments of cognitions and emotions were administered ~50 min apart. Additionally, it should be noted that we also collected saliva samples at four timepoints for future analysis of biomarkers associated with elevation.^1^

Forty-six participants were enrolled, but five participants were excluded because they did not complete the experimental tasks. One participant was removed because study staff noted significant issues with inattention throughout the session (e.g., sleeping during videos), one participant was removed because of technical issues that prevented them from completing the second half of the session, and one participant was removed for having 87.5% of their data missing. All remaining participants had complete data except three veterans who had one item missing across three separate measures. The final sample included 38 veterans. Descriptive statistics for demographic characteristics and military history are reported in Table 1. The most commonly endorsed trauma was the sudden death of a friend/loved one (94.74%) and the most common *worst trauma* was related to combat (55.26%). The means and standard deviations for all measures used in subsequent analyses are reported in Table 2.

**Table 1:** Demographic characteristics and military history of study sample.

**Variable**	***M* (*SD*) or *n* (%)**
Age	57.97 (11.96)
Gender	
Male	30 (78.95%)
Female	7 (18.42%)
Transgender (Male to Female)	1 (2.63%)
Race^*^	
White	25 (65.79%)
Black or African American	8 (21.05%)
American Indian or Alaska Native	3 (7.89%)
Other	2 (5.26%)
Native Hawaiian or Pacific Islander	1 (2.63%)
Hispanic	5 (13.16%)
Military branch^*^	
Army	28 (73.68%)
Navy	5 (13.16%)
Marine Corps	4 (10.53%)
Air Force	1 (2.63%)
National Guard	1 (2.63%)
Combat exposure? (Yes/No)	30 (78.95%)
Number of deployments	3.08 (1.73)

* *Race and military branches were not mutually exclusive and participants were allowed to select all that apply*.

**Table 2:** Mean and standard deviation for study variables at all timepoints.

**Variable**	**Baseline**	**State responses**	**T1: Post-trauma narrative**	**T2: Post-elevation videos**
PCL-5 Total	58.13 (10.68)			
Re-experiencing	14.20 (3.48)			
Avoidance	6.89 (1.25)			
Neg cognition/mood	19.68 (4.49)			
Hyperarousal	17.37 (3.85)			
State elevation–Video 1		20.47 (11.23)		
State elevation–Video 2		21.75 (12.12)		
Average state elevation		21.11 (11.29)		
State PA–Video 1		9.00 (4.88)		
State PA–Video 2		9.63 (5.09)		
Average state PA		9.32 (4.72)		
State shame			12.55 (5.94)	9.54 (4.67)
State guilt			13.95 (6.16)	11.08 (5.56)
PTCI Negative views of self			79.61 (27.02)	68.34 (27.46)
PTCI Self-blame			15.76 (8.18)	14.39 (7.74)
PTCI Negative views of world			38.17 (7.23)	34.97 (8.74)

*N = 38; PCL-5, Post-traumatic Checklist for DSM-5; PA, positive affect; PTCI, Post-traumatic Cognitions Inventory*.

### Measures

#### PTSD Symptoms

The 20-item PCL-5 (36) assessed the severity of PTSD symptoms over the past month. Items were rated on a Likert-style scale from 1 (*not at all*) to 5 (*extremely*). Total scores are calculated by summing all items with higher scores indicating greater symptom severity. In this sample, person mean imputation was used to create total scores because one participant had one missing item. The PCL-5 has demonstrated adequate reliability and validity in previous studies that included veteran samples, with evidence that scores of 33 or higher indicate subclinical or clinical PTSD severity (37, 38). Internal consistency in the present sample was α = 0.83 [95% CI: 0.76, 0.91].

#### Trauma History

The Trauma Life Events Questionnaire [TLEQ; (39)] assessed history of trauma exposure by asking participants to indicate whether they experienced any of 24 different traumatic events. Examples of traumatic events assessed include natural disasters, motor vehicle accidents, warfare or combat, and sexual assault. Participants also indicated the worst traumatic event by selecting the trauma type that “causes the most distress.” This measure was included to characterize the sample.

#### Video Responses

##### State Elevation

Currently, there are no standardized measures for assessing state-level elevation. The Elevation Scale [ES; (40)] or a modified version of it is widely used, but this scale assesses trait-like tendencies to experience elevation rather than a momentary state response. To capture state elevation in this study (i.e., response to elevation stimuli), we combined items adapted from the ES with items from a daily elevation measure previously used with a clinical population (41). Instructions from the ES were altered by asking participants to rate the extent they experienced 12 items immediately after watching elevation video stimuli on a scale from 0 (*not at all*) to 4 (*extremely*). Example items include “Somehow ‘lifted up’ or in touch with the better parts of myself,” “Tears in my eyes,” and “Motivated to live in a nobler or virtuous way” (see Supplementary Material for full measure). All items were summed to create a total score using person mean imputation because one participant had one missing item. Higher scores indicated greater elevation. Internal consistency in the present sample was α = 0.94 [0.92, 0.97] for state elevation following Video 1 and α = 0.95 [0.93, 0.97] for state elevation following Video 2.

##### Positive Affect

The Positive Affect and Negative Affect Schedule [PANAS; (42)] assessed positive affect (PA) in response to session tasks, including elevation videos. Five items were rated on a scale from 1 (*very slightly or not at all*) to 5 (*extremely*). Example items include “alert” and “inspired.” Total scores were computed by summing all five items. Higher scores indicated greater positive affect. Internal consistency for state PA was α = 0.87 [0.81, 0.93] following Video 1 and α = 0.89 [0.85, 0.92] following Video 2.

#### Pre-post Measures

##### Shame and Guilt

The 10-item State Shame and Guilt Scale [SSGS; (43)] assessed severity of state-level shame and guilt at T1 and T2. Items were rated on a scale from 1 (*I do not feel this way at all*) to 5 (*I feel this way very strongly*) and summed to create total scores, with higher scores indicating more severe shame and guilt. Example items for shame include “I feel that I am a bad person” and “I feel worthless, powerless,” and example items for guilt include “I feel remorse, regret” and “I feel like apologizing, confessing.” Internal consistency for state shame was α = 0.88 [0.82, 0.94] at T1 and α = 0.90 [0.84, 0.95] at T2; state guilt was α = 0.86 [0.79, 0.94] at T1 and α = 0.90 [0.85, 0.95] at T2.

##### Negative Trauma-Related Cognitions

The 36-item Posttraumatic Cognition Inventory [PTCI; (44)] assessed strength of negative cognitions and beliefs associated with an index trauma—in this case, the index trauma referred to the trauma described in the written narrative exercise. Each item is rated from 1 (*totally disagree*) to 7 (*totally agree*). Administration for this study was modified by instructing veterans to rate the extent to which they endorsed each item “at this moment.” The PTCI was administered twice: at T1 and T2. Scores on each item are summed to create subscale scores for negative views of the self (e.g., “I am inadequate”), the world or others (e.g., “People can't be trusted”), and self-blame (e.g., “The event happened because of the way I acted”), with higher scores indicating stronger beliefs. Person mean imputation was used for calculating subscale scores given that one participant had one missing item. Previous studies demonstrated reliability and validity for all three subscales (45). This study also found adequate reliability for each subscale: PTCI negative view of self was α = 0.95 [0.93, 0.98] at T1 and α = 0.96 [0.94, 0.98] at T2; PTCI negative view of others was α = 0.80 [0.70, 0.90] at T1 and α = 0.88 [0.82, 0.94] at T2; PTCI self-blame was α = 0.87 [0.80, 0.94] at T1 and α = 0.86 [0.79, 0.94] at T2.

### Data Analytic Plan

All data management and analyses were conducted with R (46). First, because the amount of missingness is limited to three items across the final sample and appears to be completely random (distinct participants and measures), missing data was handled with person mean imputation for the three measures that include one missing item each. Next, to test our hypothesis that emotions and cognitions reduced from T1 to T2, we used the base stats package to calculate paired samples *t*-tests and the rstatix package (47) to estimate standardized mean difference effect sizes with 95% confidence intervals (CI). We interpreted *d* > 0.20, *d* > 0.50, and *d* > 0.80 as small, medium, and large effects (48).

To test our second hypothesis that higher elevation experienced after videos would be associated with greater reductions in cognition and emotion, we used a series of multiple linear regression models for five outcomes of interest: guilt, shame, negative views of self, self-blame, and negative views of others. Specifically, we fit a baseline model and a theoretical model for each outcome in which the T2 score of that cognition or emotion was set as the dependent variable. The baseline model included the T1 score of the same target variable such that remaining variance explained in the model could be attributed to residual change in that variable. We also added the average PA score across both videos as a covariate in the baseline model to account for the potential variance explained by PA (i.e., generic, positive emotional response). The theoretical model added the average elevation score across both videos as a predictor in addition to the T1 score and average PA score as covariates. Thus, effects for average elevation as a predictor would demonstrate the extent to which elevation responses explained unique variance in the observed residual change or decrease for each outcome above and beyond the effects of PA. Regression coefficients were reported in the standardized metric (β).

We also examined *R*^2^ to understand the percent of variance accounted for in the model and inspected *f*^2^ to assess effect size. To further examine the hypothesis that higher elevation is linked with greater reductions in trauma-related cognitions and emotions, we calculated changes in effect sizes (Δ*f*^2^) between the baseline model and the theoretical model after average state elevation was added as a predictor. We interpreted changes in *f*^2^ > 0.02, *f*^2^ > 0.15, and *f*^2^ > 0.35 as small, medium, and large effects (48) attributable to the addition of state elevation. We also calculated 95% CI for change in *f*^2^ using online calculators (49).

## Results

### Changes in Cognitions and Emotions

Consistent with hypotheses, participants reported medium significant decreases in state guilt and state shame from the moment they completed the trauma narrative exercise (T1) to after viewing both videos and completing the reflection exercise (T2; see Table 3). Regarding PTCs, consistent with hypotheses, participants reported medium significant decreases in negative beliefs about self from pre- to post-video, and small significant decreases in self-blame and negative beliefs about others.

**Table 3:** Comparing cognitions and emotions between post-trauma narrative and post-elevation induction.

**Variable**	***M*_diff_**	***t***	***p***	***d* (95% CI)**
Guilt (SSGS)	−2.87	4.21	<0.001	0.68 [0.48, 0.98]
Shame (SSGS)	−3.11	3.71	<0.001	0.60 [0.43, 0.85]
Negative beliefs about self (PTCI)	−11.26	4.23	<0.001	0.69 [0.47, 0.96]
Self-blame (PTCI)	−1.37	2.21	0.033	0.36 [0.01, 0.72]
Negative beliefs about others/world (PTCI)	−3.19	2.81	0.008	0.46 [0.21, 0.69]

*N = 38; Based on Cohen (48), d > 0.20, d > 0.50, and d > 0.80 are interpreted as small, medium, and large effects. SSGS, State Shame and Guilt Scale; PTCI, Post-traumatic Cognitions Inventory*.

### The Impact of Elevation on Changes in Cognitions and Emotions

As hypothesized, higher elevation following the two elevation-inducing videos and reflection exercise was a significant predictor of residual change or decrease in state shame (β = −0.40, *SE* = 0.19, *p* = 0.038) from before the elevation videos, above and beyond the effects of positive affect experienced in response to the videos. Furthermore, adding elevation as a predictor of change resulted in a medium-sized contribution to the model for state shame (Δ*f*^2^ = 0.21), with the theoretical model explaining 42% of the variance in shame at T2. However, contrary to hypotheses, higher elevation was not a significant predictor of residual change for guilt (β = −0.24, *SE* = 0.16, *p* = 00.139). The addition of state elevation resulted in a medium-sized contribution to the guilt model (Δ*f*^2^ = 0.18), but the 95% CI included zero (−0.03, 0.52). Since state elevation was not a significant predictor in the model by itself, the medium-sized effect should be interpreted with caution. See Table 4 for a summary of models.

**Table 4:** Model comparison for baseline and theoretical models (IV = state elevation average; COV = T1 score, PA Ave; DV = T2 score).

**Residual change**	**Baseline model *F* and *R*^2^**	**Elevation model *F* and *R*^2^**	**β (*SE*)**	**Δ*f*^2^ [95% CI]**
Guilt (SSGS)	*F*_(2, 35)_ = 24.39, *p* < 0.001, *R*^2^ = 0.58	*F*_(3, 34)_ = 17.62, *p* < 0.001, *R*^2^ = 0.61	−0.24 (0.16)	0.18 [-0.03, 0.52]
Shame (SSGS)	*F*_(2, 35)_ = 8.93, *p* < 0.001, *R*^2^ = 0.34	*F*_(3, 34)_ = 8.12, *p* < 0.001, *R*^2^ = 0.42	–0.40 (0.19)^*^	**0.21 [-0.02, 0.59]**
Neg view of self (PTCI)	*F*_(2, 35)_ = 35.59, *p* < 0.001, *R*^2^ = 0.67	*F*_(3, 34)_ = 28.93, *p* < 0.001, *R*^2^ = 0.72	–0.33 (0.14)^*^	**0.54 [0.15, 1.32]**
Self-blame (PTCI)	*F*_(2, 35)_ = 64.52, *p* < 0.001, *R*^2^ = 0.79	*F*_(3, 34)_ = 41.85, *p* < 0.001, *R*^2^ = 0.79	0.02 (0.11)	0.00 [0.00, 0.00]
Neg view of others (PTCI)	*F*_(2, 35)_ = 12.20, *p* < 0.001, *R*^2^ = 0.41	*F*_(3, 34)_ = 10.61, *p* < 0.001, *R*^2^ = 0.48	–0.38 (0.17)^*^	**0.23 [-0.01, 0.63]**

* *p < 0.05; Based on Cohen (48), f^2^ ≤ 0.02 was interpreted as a small effect; f^2^ ≤ 0.15 as a medium effect; and, f^2^ ≤ 0.35 as a large effect. SSGS, State Shame and Guilt Scale; PTCI, Post-traumatic Cognitions Inventory. The Bold face and asterisk indicates p < 0.05*.

As hypothesized, after accounting for positive affect experienced in response to videos, higher elevation was also a significant predictor of residual change or decrease in negative beliefs about self (β = −0.33, *SE* = 0.14, *p* = 0.022) and negative beliefs about others (β = −0.38, *SE* = 0.17, *p* = 0.036). The addition of elevation resulted in a large-sized contribution to the model for negative beliefs about self (Δ*f*^2^ = 0.54), with the theoretical model explaining 72% of the variance explained in negative views of self at T2. Furthermore, adding elevation as a predictor of change in negative beliefs about others resulted in a medium-sized contribution to the model (Δ*f*^2^ = 0.23) with the theoretical model explaining 48% of the variance in negative views of others at T2. However, contrary to hypothesis, higher elevation was not a significant predictor of self-blame (β = 0.02, *SE* = 0.11, *p* = 0.833). The addition of state elevation did not contribute significant changes in the effect size of the self-blame model (Δ*f*^2^ = 0.00).

## Discussion

Existing literature supports the notion that veterans with PTSD can experience elevation and that doing so may elicit distinct responses from other positive emotions. The purpose of the present study was to further evaluate the role of elevation on trauma-related cognitions and emotions among a sample of veterans with probable PTSD. It was hypothesized that negative emotions (shame and guilt) and negative cognitions about the self, others, and self-blame would reduce from T1 to T2. We also hypothesized that higher levels of elevation reported following exposure to elevation-inducing videos would predict greater decreases in negative emotions and cognitions from post-trauma narrative to post-elevation induction.

### Changes in Cognitions and Emotions

Consistent with hypotheses, there were significant differences in levels of negative emotions (i.e., shame and guilt) and negative cognitions about the self, others, and self-blame between T1 to T2; these reduced from after a written trauma narrative exercise to after watching and journaling about elevating videos. Pre-post mean difference scores ranged in magnitude from small to medium, with the largest difference found in participants' negative views about the self, followed by guilt, shame, and negative beliefs about others. Self-blame had the smallest effect relative to other changes, which could suggest that guilt, shame, and the other types of trauma cognitions may be particularly amenable to change following exposure to elevation stimuli, whereas self-blame may be less flexible or more challenging to modify. One possible explanation for the smaller effect is potential heterogenous experiences of self-blame. For example, Janoff-Bulman (50) proposed two types of self-blame attributions: behavioral and characterological self-blame. Behavioral self-blame reflects survivors' belief that their own behavior led to the traumatic event. Characterological self-blame focuses on the survivor's personality or character as the cause of event, rather than their behavior. Perhaps it is difficult to modify specific self-blame attributions, which could be made even more rigid depending on the type of traumatic event (e.g., behavioral self-blame for engaging in combat). More research is needed to understand how variations of self-blame and the context of traumatic events influences changes in beliefs about self-blame, particularly to the extent it informs existing and future treatment application.

### Effects of Elevation on Changes in Cognitions and Emotions

Subsequent analyses were used to expand on these findings by assessing the extent to which reported levels of elevation predicted changes in each cognition and emotion. As hypothesized, higher state elevation experienced after viewing inspirational videos and completing a reflection exercise was a significant predictor of reductions in shame, negative views of self, and negative views of others. Regarding significant findings, there was a medium effect on changes in shame and negative views of others, which should be interpreted with caution given that the lower bounds of the 95% confidence intervals were slightly below zero (shame LLI = −0.02; neg view of others LLI = −0.01). On the other hand, we found a large effect for negative views of the self. Notably, these findings reflect the unique association with elevation experienced during the videos above and beyond the effects of general PA. Although we did not directly test mechanisms for these associations, there are several potential explanations based on the existing literature for these outcome variables.

Shame is an affective state that includes a focus and concern about the self; negative experiences are a reflection of the *bad self* and the self is consequently scrutinized and negatively evaluated (51). Given that elevation is theorized to shift attention outside the self (52), it may impact levels of shame by redirecting one's focus from negative self-perceptions to the uncommon acts of goodness or moral beauty of others. Shame is characterized by heightened self-focus, whereas elevation is an other-praising emotion that draws attention toward the good in others (8, 14).

Because elevation involves positive appraisals of others' actions by definition, it follows that there would be a significant effect on changes in negative cognitions about the world. Specifically, negative cognitions about the world involve beliefs that people cannot be trusted and that the world is a dangerous place (44), whereas experiences of elevation are associated with connectedness, openness, and loving feelings toward other people [e.g., (40)]. Perhaps elevation provides an opportunity to collect evidence that might contradict strong negative beliefs about others, while also providing exposure to experiencing genuine compassion toward strangers or humanity in general. These findings are consistent with another study that examined daily elevation in a clinical population of people with depression and anxiety, which suggested higher levels of elevation were linked to stronger beliefs that people should look out for one another, perceived closeness to others, and urges to support and help people (41).

Although elevation is an *other*-praising emotion, it was also associated with a large magnitude change in negative views of the self. Notably, this was a stronger effect relative to medium changes in negative views of others. One potential explanation for this finding is that elevation might increase awareness of one's capacity for good or moral characteristics in general. Monin (53) proposed that observing exceptional moral behavior may trigger an upward social comparison, which might remind people of their own values and elicit evidence or recognition that one is capable of acting in line with those values [e.g., being a good, moral person; (54)]. A study by Schnall and Roper (12) also found that self-affirmation (before an elevating film clip) was associated with participants' identifying positive qualities within themselves (e.g., helping others) and accessing evidence of their own ability to do good. Lastly, previous studies have found that elevation is associated with a strong desire to become a better person [e.g., (55)]; perhaps experiencing the motivation for self-improvement alone has some kind of positive impact on views of the self. Now that the present study provides initial evidence that elevation is associated with immediate cognitive and emotional changes, future research is needed to expand on these findings and decipher the specific mechanisms that facilitate the change process, and the extent to which these immediate changes have any kind of long-term, positive impact on trauma recovery.

Contrary to hypotheses, state elevation was not a significant predictor of changes in guilt or self-blame. There are several possible reasons this association was not observed in the present study. First, regarding self-blame, it could be related to the smaller reduction in scores from T1 to T2, which may indicate there was not enough variance or change in reported self-blame for state elevation to be a meaningful predictor above and beyond the effects of PA. Alternatively, guilt and self-blame might be conceptually different than the other outcomes in ways that might be differentially influenced by elevation. For example, guilt and self-blame share some conceptual features insofar as they could be more tied to specific facts surrounding certain events/trauma that might be more descriptive of the traumatic incident itself (56) and less flexible to interpretation (e.g., I did not fight-off my attacker). Alternatively, shame and negative cognitions of the self/world could be described as more generalized and somewhat based on interpretations of the traumatic event or one's actions/inaction; therefore, those generalized beliefs or interpretations may be more amenable to change than guilt or self-blame. For example, it seems possible that elevation may not impact guilt or self-blame if a veteran believes they had some degree of responsibility for an event (e.g., I had a role in a friend's death during a combat situation), but one can reasonably assume it is possible to reduce the amount of shame that veteran experiences as a result of that same event. Further research is needed to identify mechanisms exclusive to changes in guilt and self-blame, and to determine whether features of these symptoms are in less likely to be modified following experiences of elevation.

### Potential Treatment Implications

These findings have potential implications for the clinical utility of elevation and treatment for trauma distress. First, it should be noted that the long-term effects of inducing elevation are unknown and beyond the scope of this study. These findings primarily support an initial proof of concept that elevation may play a role in immediate or momentary shifts in relevant cognitions and emotions, above and beyond the effects of experiencing general PA. Accordingly, in the short-term, elevation might be useful as a novel tool to regulate emotions and buffer against strong negative beliefs in a given moment that veterans with PTSD encounter in their daily life. For example, it might be helpful in response to distress associated with trauma reminders, similar to this study in which elevation induction occurred immediately after a trauma-cue reminder. The extent to which momentary shifts *via* elevation promote long-term changes in symptoms and behaviors is unknown; thus, future research is needed to better understand the clinical utility of these immediate elevation reactions.

If additional empirical support is found, one example of how experiencing elevation could have long-term implications is by promoting increased trait-like sensitivity to experiencing elevation through repeated elevation inductions, as indicated in past work with civilian, non-clinical populations [e.g., (14)]. The potential clinical utility of a greater predisposition toward experiencing elevation could include a greater awareness of the goodness in others and increased likelihood of feeling motivated to engage in behaviors consistent with trauma recovery (e.g., connect with others, pro-sociality). If elevation is to be used as a therapeutic tool, then more research is required to better understand the additive and long-term effects of experiencing momentary states of elevation, determine whether elevation *via* exposure to others' virtuous behavior leads to observable mechanisms as theorized (e.g., increased social engagement), and assess whether there are identifiable mechanisms that directly mediate decreased PTSD symptoms and increased well-being. Furthermore, implementation research is also needed to examine whether elevation induction and related activities can be used as a brief skill or exercise, a standalone intervention, or perhaps a supplement to other treatments for PTSD.

### Limitations

The findings of the present study should be considered in the light of study limitations. First, we used a small sample size that was primarily male and White. Future studies should aim to expand our understanding of this phenomenon with larger and more diverse samples. Related, because the authors are unaware of other studies examining elevation and trauma-related symptoms in an experimental study, it is unclear if these findings are specific to veterans or if they generalize to the civilian population, and subsequently a wider-range of trauma types. Given that there were no standardized measures for assessing state elevation when this study was conducted, the elevation measure we used is limited and further psychometric analyses are needed. Without developing and testing a new measure, we aimed to address this limitation by utilizing an elevation measure that demonstrated adequate reliability in past studies (40), is consistent with the established theoretical underpinnings of elevation, and demonstrated high internal consistency in the present study. Next, the present study examined change in several outcomes following elevation stimuli alone without a control condition. This decision was based on resource constraints and previous findings that elevation responses were distinct following the elevation videos compared to amusement videos, which suggests the same video stimuli used in this study would elicit the desired emotional response. However, the lack of a control group in the present study that examined changes in different outcomes precludes causal inference. Lastly, there are additional, unaccounted features of the study design that could have contributed to changes in pre-post scores. For example, because the journal exercise (i.e., reflecting on the elevation videos) was completed before the administration of T2 measures, participating in the journal exercise could have influenced the self-reported reduction of cognitions and emotions above and beyond the effects of state elevation following the videos—the primary predictor of change in our analyses. Improvements in cognition and emotion scores could have also been influenced by other features like generalized distraction from thinking about the traumatic event, return to baseline after activating trauma-related distress (i.e., regression to the mean), or unintended demand characteristics of the procedure. For example, high levels of social desirability could also influence reported changes as well as state elevation responses; thus, further research is needed to fully assess potential confounding variables with an appropriate sample size. Overall, future studies may benefit by expanding to include a control condition and aim to differentiate the effects of various components of engagement with virtuous behavior to determine specific mechanisms that might drive changes in trauma-related symptoms.

## Conclusion

Findings from this study showed significant changes in negative emotions and cognitions about the self, others, and self-blame following a written trauma narrative and elevation-inducing videos. Greater experiences of elevation after watching elevation-inducing videos were associated with shame, negative cognitions about the self, and negative cognitions about others. According to results, exposure to elevation videos may shift trauma-related cognitions and emotions among a sample of veterans with probable PTSD. Future studies should continue to explore this positive emotion and expand our understanding of the clinical utility of elevation.

## Data Availability Statement

The data presented in this article is not readily available because given the nature of this research, participants of this study did not agree for their data to be shared publicly. Requests to access the data should be directed to adam.mcguire@va.gov.

## Ethics Statement

This study involved human participants and was reviewed and approved by Central Texas Veterans Health Care System Institutional Review Board. The patients/participants provided their written informed consent to participate in this study.

## Author Contributions

AM, BH, and YS collected data. AM analyzed the data with statistical consultation from YS. AM, JF, BH, AW, and YS drafted the manuscript. BH, JF, and AW provided material support. BH provided administrative and technical support. All authors provided critical revision of the manuscript for important intellectual content and read, discussed, and approved the final manuscript.

## Funding

AM was supported by a Small Projects in Rehabilitation Research Award I21-RX003035 and YS was supported by Career Development Award IK1-RX003122 from the United States (U.S.) Department of Veterans Affairs, Rehabilitation Research and Development Service.

## Author Disclaimer

The views expressed herein are those of the authors and do not necessarily reflect the official policy or position of the Department of Veterans Affairs or the United States Government.

## Conflict of Interest

The authors declare that the research was conducted in the absence of any commercial or financial relationships that could be construed as a potential conflict of interest.

## Publisher's Note

All claims expressed in this article are solely those of the authors and do not necessarily represent those of their affiliated organizations, or those of the publisher, the editors and the reviewers. Any product that may be evaluated in this article, or claim that may be made by its manufacturer, is not guaranteed or endorsed by the publisher.

## Footnotes

^1^Participants completed additional measures that were added for secondary analyses and not reported here. A list of all measures and the administration schedule can be found in the Supplementary Material.
