# Supplementary material for: Changes in Trauma-Related Cognitions and Emotions After Eliciting Moral Elevation: Examining the Effects of Viewing Others' Virtuous Behavior on Veterans With PTSD
Source: Front Health Serv. 2022 Feb 4;1:831032. doi: 10.3389/frhs.2021.831032 (PMC9009273; doi:10.3389/frhs.2021.831032)
Supplement: Supplementary file 1 [file Data_Sheet_1.docx]

Changes in Trauma-Related Cognitions and Emotions After Eliciting Moral Elevation: Examining the Effects of Viewing Others’ Virtuous Behavior on Veterans with PTSD

**Supplemental Materials**

1. Video links
   1. Neutral video:
      1. How Flutes Are Made: <https://youtu.be/YqT5tok8ISo>
   2. Elevation videos:
      1. Subway Hero: <https://youtu.be/cKuWjZe4q20>
      2. Team Hoyt: <https://youtu.be/2vmeE3OCGs0>

| **Table 1. Assessment Instruments** | | | | | | | |
| --- | --- | --- | --- | --- | --- | --- | --- |
| **Measure** | **# Items** | **T1** | **T2** | **T3** | **T4.1** | **T4.2** | **T5** |
| *PTSD Checklist for DSM-5 (PCL-5)^1^ | 20 |  |  |  |  |  |  |
| International Positive and Negative Affective Scale – Short Form (I-PANAS-SF)^11^ | 10 | X | X | X | X | X |  |
| State Shame and Guilt Scale (SSGS)^12^ | 10 | X | X |  |  |  | X |
| Demographic Questionnaire and Military History Form | 20 | X |  |  |  |  |  |
| Saliva Questionnaire (SQ)^13^ | 11 | X |  |  |  |  |  |
| Alcohol Use Disorders Identification Test (AUDIT)^14^ | 10 | X |  |  |  |  |  |
| Drug Abuse Screening Test (DAST)^15^ | 10 | X |  |  |  |  |  |
| Traumatic Life Events – Veteran Version (TLEQ)^16^ | 24 | X |  |  |  |  |  |
| The Brief Traumatic Brain Injury Screen (BTBIS)^17^ | 3 | X |  |  |  |  |  |
| Marlowe-Crown Social Desirability Scale 10-Item Short Form (M-CSDS-10)^18^ | 10 | X |  |  |  |  |  |
| Freiburg Mindfulness Inventory (FMI) – Shortened Version^2^ | 14 | X |  |  |  |  |  |
| Engagement with Beauty Scale (EBS)^3^ | 6 | X |  |  |  |  |  |
| Gratitude Questionnaire-6 (GQ-6)^4^ | 6 | X |  |  |  |  |  |
| Patient Health Questionnaire (PHQ-9)^5^ | 9 | X |  |  |  |  |  |
| Critical Warzone Experiences Scale (CWE)^6^ | 7 | X |  |  |  |  |  |
| Brief Resilience Scale (BRS)^7^ | 6 | X |  |  |  |  |  |
| Quality of Life Scale (QOLS)^8^ | 16 | X |  |  |  |  |  |
| Posttraumatic Cognitions Inventory (PTCI)^9^ | 33 |  | X |  |  |  | X |
| Moral Injury Events Scale (MIES)^10^ | 9 |  | X |  |  |  | X |
| Expressions of Moral Injury Scale (EMIS-M)^19^ | 17 |  | X |  |  |  | X |
| Brief Emotion Rating Questionnaire | 16 |  |  | X | X | X |  |
| Rumination Item Question | 2 |  |  |  |  |  | X |
| Video Questionnaire | 2 |  |  |  |  |  | X |

*PCL-5 was administered at prescreen prior to baseline measures.

1. Written Trauma Narrative Instructions

*“THIS PAPER WILL NOT BE TURNED IN TO THE RESEARCH ASSISTANT AND IS FOR YOUR EYES ONLY. PLEASE HOLD ON TO THIS PAPER AND TAKE IT WITH YOU TO DESTROY OR DO WHATEVER YOU WISH. DO NOT WRITE YOUR NAME ON THIS PAPER.*

*Please take the next 15 minutes to write an account of your most distressing traumatic event in the space below. Please try to include as many sensory details (sights, sounds, smells, etc.) as possible. Also, include as many of your thoughts and feelings that you recall having during the event.”*

1. Journal Response Instructions

*“Please take some time to reflect on the last two videos you watched.*

*Consider what you noticed in the videos and how they made you feel. After taking some time to reflect, use the space below to describe your reaction to the videos (type in the open space). You can use the following questions as prompts:*

- *In the videos, what stood out the most to you? And why?*
- *Thinking about your own personal reaction to the videos, what changes in yourself did you notice most?*
  - *What physical and/or emotional reaction did you have to the videos?*
- *After watching the videos, what do you feel like doing?*
  - *What is something (general or specific) you feel more inclined to do after watching these videos?”*

1. References
2. Weathers, F. W., Litz, B. T., Keane, T. M., Palmieri, P. A., Marx, B. P., & Schnurr, P. P. (2013). The PTSD checklist for DSM-5 (PCL-5). https://doi.org/Scale available from the National Center for PTSD at www.ptsd.va.gov
3. Walach, H., Buchheld, N., Buttenmüller, V., Kleinknecht, N., & Schmidt, S. (2006). Measuring mindfulness—the Freiburg mindfulness inventory (FMI). *Personality and individual differences*, *40*(8), 1543-1555. https://doi:10.1016/j.paid.2005.11.025.
4. Diessner, R., Solom, R. D., Frost, N. K., Parsons, L., & Davidson, J. (2008). Engagement with beauty: Appreciating natural, artistic, and moral beauty. *The Journal of psychology*, *142*(3), 303-332. https://doi:10.3200/JRLP.142.3.303-332.
5. McCullough, M. E., Emmons, R. A., & Tsang, J. A. (2002). The grateful disposition: A conceptual and empirical topography. *Journal of personality and social psychology*, *82*(1), 112. https://doi:10.1037/0022-3514.82.1.112.
6. Kroenke, K., & Spitzer, R. L. (2002). The PHQ-9: a new depression diagnostic and severity measure. *Psychiatric annals*, *32*(9), 509-515. https://doi:10.3928/0048-5713-20020901-06.
7. Kimbrel, N. A., Evans, L. D., Patel, A. B., Wilson, L. C., Meyer, E. C., Gulliver, S. B., & Morissette, S. B. (2014). The Critical Warzone Experiences (CWE) Scale: Initial psychometric properties and association with PTSD, anxiety, and depression. *Psychiatry research*, *220*(3), 1118-1124. https://doi:10.1016/j.psychres.2014.08.053.
8. Smith, B. W., Dalen, J., Wiggins, K., Tooley, E., Christopher, P., & Bernard, J. (2008). The brief resilience scale: assessing the ability to bounce back. *International journal of behavioral medicine*, *15*(3), 194-200. https://doi:10.1080/10705500802222972.
9. Burckhardt, C. S., Woods, S. L., Schultz, A. A., & Ziebarth, D. M. (1989). Quality of life of adults with chronic illness: a psychometric study. *Research in nursing & health*, *12*(6), 347-354. https://doi:10.1002/nur.4770120604.
10. Foa, E. B., Ehlers, A., Clark, D. M., Tolin, D. F., & Orsillo, S. M. (1999). The posttraumatic cognitions inventory (PTCI): Development and validation. *Psychological assessment*, *11*(3), 303. https://doi:10.1037/1040-3590.11.3.303.
11. Nash, W. P., Marino Carper, T. L., Mills, M. A., Au, T., Goldsmith, A., & Litz, B. T. (2013). Psychometric evaluation of the moral injury events scale. *Military Medicine*, *178*(6), 646-652. https://doi:10.7205/MILMED-D-13-00017.
12. Thompson, E. R. (2007). Development and validation of an internationally reliable short-form of the positive and negative affect schedule (PANAS). *Journal of cross-cultural psychology*, *38*(2), 227-242. https://doi.org/10.1177%2F0022022106297301
13. Marschall, D., Sanftner, J., & Tangney, J. P. (1994). The state shame and guilt scale. *Fairfax, VA: George Mason University*.
14. Szabo, Y. Z., Fernandez-Botran, R., & Newton, T. L. (2019). Cumulative trauma, emotion reactivity and salivary cytokine levels following acute stress in healthy women. *Anxiety, Stress, & Coping*, *32*(1), 82-94. https://doi.org/10.1080/10615806.2018.1524377
15. Babor, T. F., Higgins-Biddle, J. C., Saunders, J. B., & Monteiro, M. G. (2001). The alcohol use disorders identification test (AUDIT): Guidelines for use in primary care. *World Health Organization, Department of Mental Health and Substance Abuse*.
16. Skinner, H. A. (1982). The drug abuse screening test. *Addictive Behaviors, 7*, 363-371.
17. Kubany, E. S., Leisen, M. B., Kaplan, A. S., Watson, S. B., Haynes, S. N., Owens, J. A., & Burns, K. (2000). Development and preliminary validation of a brief broad-spectrum measure of trauma exposure: the Traumatic Life Events Questionnaire. *Psychological assessment, 12*(2), 210. https://doi.org/10.1037/1040-3590.12.2.210
18. Schwab, K., Baker, G., Ivins, B., Sluss-Tiller, M., Lux, W., & Warden, D. (2006). The Brief Traumatic Brain Injury Screen (BTBIS): Investigating the Validity of a Self-report Instrument for Detecting Traumatic Brain Injury (TBI) in Troops Returning from Deployment in Afghanistan and Iraq. *Neurology, 66(*5).
19. Barger, S. D. (2002). The Marlowe-Crowne affair: Short forms, psychometric structure, and social desirability. *Journal of Personality Assessment, 79*(2), 286-305. https://doi.org/10.1207/S15327752JPA7902_11
20. Currier, J. M., Farnsworth, J. K., Drescher, K. D., McDermott, R. C., Sims, B. M., & Albright, D. L. (2018). Development and evaluation of the Expressions of Moral Injury Scale—Military Version. *Clinical Psychology & Psychotherapy, 25*(3), 474-488. https://doi.org/10.1002/cpp.2170
